# The Janus kinase 1/2 inhibitor baricitinib reduces biomarkers of joint destruction in moderate to severe rheumatoid arthritis

**DOI:** 10.1186/s13075-020-02340-7

**Published:** 2020-10-12

**Authors:** Christian S. Thudium, Anne C. Bay-Jensen, Suntara Cahya, Ernst R. Dow, Morten A. Karsdal, Alisa E. Koch, Wenling Zhang, Robert J. Benschop

**Affiliations:** 1 Nordic Bioscience, Biomarkers and Research, Herlev Hovedgade 205-207, DK-2730 Herlev, Denmark; 2 Eli Lilly and Company, Indianapolis, Indiana USA

**Keywords:** Biomarkers, Baricitinib, Rheumatoid arthritis

## Abstract

**Background:**

Tissue released blood-based biomarkers can provide insight into drug mode of action and response. To understand the changes in extracellular matrix turnover, we analyzed biomarkers associated with joint tissue turnover from a phase 3, randomized, placebo-controlled study of baricitinib in patients with active rheumatoid arthritis (RA).

**Methods:**

Serum biomarkers associated with synovial inflammation (C1M, C3M, and C4M), cartilage degradation (C2M), bone resorption (CTX-I), and bone formation (osteocalcin) were analyzed at baseline, and weeks 4 and 12, from a subgroup of patients (*n* = 240) randomized to placebo or 2-mg or 4-mg baricitinib (RA-BUILD, NCT01721057). Mixed-model repeated measure was used to identify biomarkers altered by baricitinib. The relationship between changes in biomarkers and clinical measures was evaluated using correlation analysis.

**Results:**

Treatment arms were well balanced for baseline biomarkers, demographics, and disease activity. At week 4, baricitinib 4-mg significantly reduced C1M from baseline by 21% compared to placebo (*p* < 0.01); suppression was sustained at week 12 (27%, *p* < 0.001). Baricitinib 4-mg reduced C3M and C4M at week 4 by 14% and 12% compared to placebo, respectively (*p* < 0.001); they remained reduced by 16% and 11% at week 12 (*p* < 0.001). In a pooled analysis including all treatment arms, patients with the largest reduction (upper 25% quartile) in C1M, C3M, and C4M by week 12 had significantly greater clinical improvement in the Simplified Disease Activity Index at week 12 compared to patients with the smallest reduction (lowest 25% quartile).

**Conclusion:**

Baricitinib treatment resulted in reduced circulating biomarkers associated with joint tissue destruction as well as concomitant RA clinical improvement.

**Trial registration:**

ClinicalTrials.gov NCT01721057; date of registration: November 1, 2012

## Background

Rheumatoid arthritis (RA) is a chronic autoimmune disease characterized by polyarticular inflammation of the joints. The inflammation is associated with synovitis, osteitis, and periarticular osteopenia. Joints exhibit the cartilage loss and bone erosions of the subchondral bone, leading to loss of joint function and disability [1, 2]. Disease progression in RA is driven largely by the effects of pro-inflammatory cytokines such as interleukin (IL)-6 and tumor necrosis factor (TNF)-alpha [2] as well as IL-21 [3], continuously secreted by immune cells in an inflammatory environment. Through activation of their receptors, these cytokines initiate signaling cascades which include the activation of downstream Janus kinase (JAK), spleen tyrosine kinase, and mitogen-activated protein kinase resulting in the increased expression of proteolytic enzymes such as matrix metalloproteinases (MMPs) [4]. The joint tissues consist of mainly collagens and collagen-binding proteins. In RA, elevated levels and activity of proteolytic enzymes, such as MMPs, lead to the generation of collagen-specific fragments, which have been targeted as blood-based biomarkers reflecting tissue turnover and destruction. Synovial inflammation is an important aspect of RA disease pathology. The type I collagen-derived fragment C1M is generated by MMPs cleaving type I collagen and has been associated with general tissue inflammation [5]. C3M, which is a peptide released following MMP-mediated cleavage of type III collagen, has been associated with inflammation-driven tissue turnover and fibrosis [6, 7]. Another marker associated with synovial inflammation, C4M, is released following MMP-mediated cleavage of type IV collagen, a major constituent in the basement membrane. Changes in this marker have previously been associated with inflammation-driven tissue turnover [8, 9]. RA affects the cartilage as well. Examples of cartilage degradation biomarkers include C2M fragments released after cleavage of type II collagen in cartilage by MMPs [10]. Finally, an important aspect of RA is bone erosions, and halting the progression of these erosions is an important treatment goal. A number of blood-based biomarkers have been developed that reflect systemic bone turnover status. Examples of such biomarkers include CTX-I, a fragment of type I collagen specifically generated by cathepsin K cleavage that reflects the rate of osteoclast-mediated bone resorption. Osteocalcin, which is largely embedded in the bone and released upon bone resorption, also marks bone turnover. However, the molecule is also produced by osteoblasts and specific forms of osteocalcin can reflect bone formation [11]. Thus, changes in serum biomarkers associated with joint tissue remodeling include synovial inflammation (C1M, C3M, and C4M), cartilage degradation (C2M), and bone resorption (CTX-I) and formation (osteocalcin).

Baricitinib is an oral selective inhibitor of JAK1 and JAK2. JAK1/2 are critical signaling components downstream of several different cytokine receptors, including interferon gamma (IFNγ) receptor, IL-6 receptor, and common γ-chain receptor family [12]. In the phase 3 study RA-BUILD (NCT01721057), once-daily baricitinib yielded significant clinical benefit in patients with active RA who had inadequate response or intolerance to conventional synthetic disease-modifying antirheumatic drugs (csDMARDs) compared to placebo [13]. In the current study, we measured changes in serum biomarkers associated with joint tissue remodeling and determined the relationship between changes in those biomarkers and clinical response.

## Methods

### Study design and patients

The study design and patient inclusion/exclusion criteria for RA-BUILD have been described previously [13]. Briefly, 684 adult patients, in RA-BUILD with active RA (≥ 6/68 tender and ≥ 6/66 swollen joints; serum high sensitivity C-reactive protein [hsCRP] ≥ 3.6 mg/L) and an insufficient response (despite prior therapy) or intolerance to ≥ 1 csDMARDs were randomized 1:1:1 to receive oral placebo or 2- or 4-mg oral baricitinib once daily. In this biomarker subanalysis, 240 patients (80 from each treatment group, based on a power analysis using a 2 sample *t* test and correlation analyses) were selected through a stratified sampling using the following key baseline variables: hsCRP, modified Total Sharp Score (mTSS), swollen joint count, physician global assessment, seropositivity for rheumatoid factor (RF), and anti-citrullinated peptide antibodies (ACPA), and tobacco use. Based on these parameters, we performed a hierarchical clustering analysis [14] and stratified the random sampling based on the resulting clusters. The objective of the stratification was to ensure that the range of values for these key baseline properties was well represented in the samples.

The study was conducted in accordance with the ethical principles of the Declaration of Helsinki and Good Clinical Practice Guidelines and was approved by the institutional review board or ethics committee at each center. All patients provided written informed consent allowing for retrospective analysis of the blood samples for assessment of biomarkers of joint tissue turnover.

### Sample processing

Exploratory serum biomarkers were assayed at baseline and weeks 4 and 12. Patients fasted prior to sample collections at baseline and week 12. Serum samples were collected and stored at − 80 °C and analyzed at the Nordic Bioscience Laboratory (Herlev, Denmark).

### Biomarker assays

The following connective tissue biomarkers were analyzed in the serum by immunoassays as described previously [15, 16]: (1) connective tissue remodeling and inflammation by C1M, C3M, and C4M (Nordic Bioscience, Herlev, Denmark); (2) cartilage degradation by C2M (Nordic Bioscience, Herlev, Denmark); and (3) bone resorption (CTX-I) and formation (osteocalcin; Roche Diagnostics, Basel, Switzerland). All markers were measured in a College of American Pathologist-certified laboratory and acceptance criteria for duplicate samples were coefficient of variation < 15%. Assay characteristics are presented in Table S1.

### Statistical analysis

The relationship between changes in biomarkers at weeks 4 and 12 (relative to baseline) and clinical response at week 12 was assessed using the following clinical measures: Hybrid American College of Rheumatology (ACR) response measure (a hybrid of ordinal and continuous versions of ACR scores) [17], ACR20/50/70% response, Clinical Disease Activity Index (CDAI), Simplified Disease Activity Index (SDAI), Health Assessment Questionnaire-Disability Index (HAQ-DI), Disease Activity Score for 28 joints with the use of the erythrocyte sedimentation rate (DAS28-ESR), and swollen and tender joint counts. We also analyzed the relationship between changes in biomarkers and radiographic scores, specifically looking at erosion, joint space narrowing, and mTSS.

**Fig. 1:**
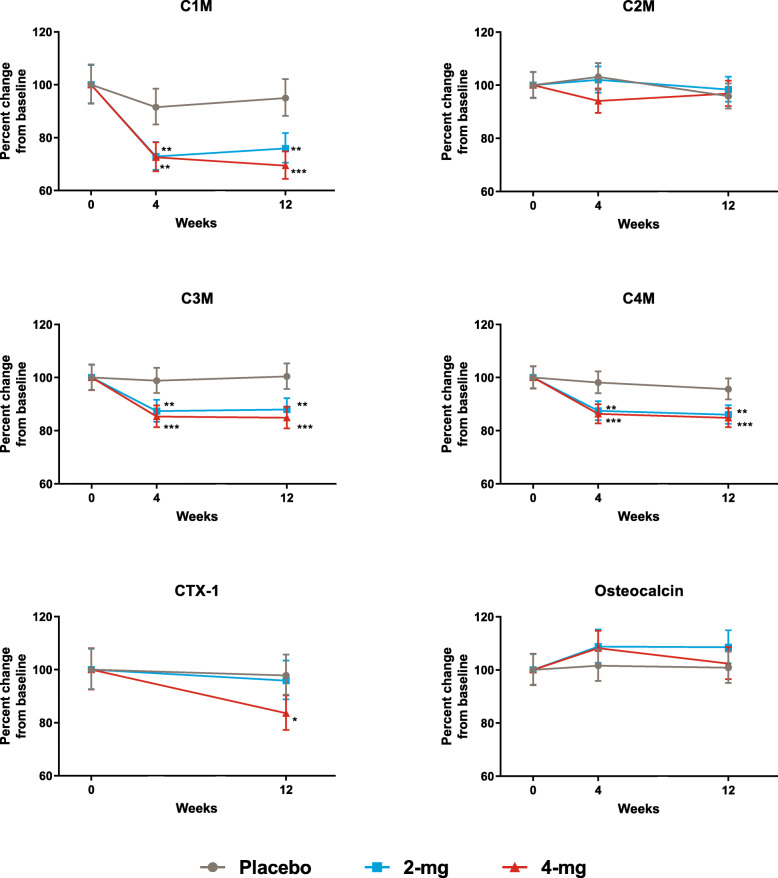
Change of biomarkers relative to baseline for all 3 treatment groups over time. Note: data for CTX-I are not presented at week 4 because patients did not fast prior to this sample collection. *p ≤ 0.05; **p ≤ 0.01; ***p ≤ 0.001 compared to placebo. *P* values are multiplicity adjusted across all potential pairwise comparisons in treatment by time using Tukey method

A mixed model repeated measure was used to identify markers that were affected by baricitinib longitudinally. The mixed model assumed compound symmetry as the covariance structure, and in addition to the main parameters of treatment group and visits, two covariates were used to normalize the geographic regions of the clinical trials and joint erosion status at baseline. A multiplicity adjustment was done using the Tukey method. The correlation analysis using the Spearman rank correlation was performed to evaluate the relationship between the observed changes in biomarkers and clinical measures at the primary endpoint of week 12. In these analyses, a log transformation was performed on the biomarker concentration levels, while all the clinical scores were not transformed. In addition to the non-parametric Spearman correlation analysis, analysis of variance was performed to evaluate the difference in clinical scores between subsets of patients with the highest and the lowest quartiles in the biomarker changes. Odds ratio was estimated as the odds of being responders, as defined by ACR endpoints (ACR20, ACR50, ACR70), for patients having a larger reduction (upper 25% quartile) in biomarkers C1M, C3M, and C4M from baseline, relative to patients having a smaller decrease (lowest 25% quartile). Except for the repeated measure analysis used for the analysis presented in Fig. 1, non-multiplicity adjusted *p* values were used.

## Results

### Study overview

Baseline characteristics and disease activity of the 240 patients included in this analysis were similar among treatment groups and had a similar distribution to the overall study population [13]; baseline concentrations and range/distribution of all biomarkers tested were similar between all three groups (Table 1).

**Table 1:** Baseline characteristics and disease activity of patients included in analysis

	Placebo (***N*** = 80)	Baricitinib 2-mg (***N*** = 80)	Baricitinib 4-mg (***N*** = 80)
Female, %	81	77	78
Rescued, %	28	3	5
ACR20 at week 12, %	37	74	66
ACR50 at week 12, %	13	41	35
BMI	29.63 (7.20)	29.31 (7.26)	29.34 (8.47)
Disease duration, years^a^	7.09 (7.98)	7.74 (7.37)	8.74 (8.01)
DAS28-ESR	6.16 (0.98)	6.25 (0.98)	6.23 (0.85)
HAQ-DI	1.46 (0.63)	1.43 (0.65)	1.49 (0.58)
Swollen joint count, of 66	12.30 (6.15)	13.67 (9.66)	13.97 (7.42)
Tender joint count, of 68	25.05 (16.55)	23.84 (14.65)	24.05 (13.89)
mTSS	15.33 (34.24)	20.05 (30.50)	26.18 (39.67)
Patient pain	57.80 (22.61)	59.53 (20.77)	55.25 (22.69)
RF, IU/mL, mean^b^ (CV)	48.71 (315.05%)	66.16 (237.16%)	56.88 (318.90%)
ACPA, U/mL, mean^b^ (CV)	64.28 (4237.03%)	71.99 (1693.91%)	69.24 (6787.05%)
Biomarkers, mean^b^ (CV)			
C1M	38.88 (72.15%)	38.95 (56.62%)	41.94 (62.12%)
C2M	0.30 (39.21%)	0.33 (42.14%)	0.33 (41.55%)
C3M	15.21 (37.42%)	16.06 (30.55%)	16.90 (41.19%)
C4M	35.69 (35.05%)	37.33 (30.12%)	38.33 (32.00%)
CRP	9.77 (153.11%)	8.86 (141.93%)	9.05 (128.21%)
CTX-I	0.30 (68.56%)	0.31 (71.17%)	0.30 (72.00%)
Osteocalcin	16.90 (49.05%)	15.93 (53.32%)	17.89 (51.33%)

Data are mean (SD) unless otherwise stated

*ACPA* anti-citrullinated peptide antibody; *ACR20/50* 20/50% improvement according to the criteria of the American College of Rheumatology; *BMI* body mass index; *CV* coefficient of variation (statistical measure of dispersion of data relative to the mean since these biomarker data follow a lognormal distribution; thus, the standard deviation does not adequately capture the nature of the dispersion of the data; *C1M* metalloproteinase-derived fragments of type I, of type II (C2M), type III (C3M), and type IV (C4M) collagen; *CRP* C-reactive protein; *CTX-I* C-terminal telopeptide of type I collagen; *DAS28* Disease Activity Score 28 joints; *ESR* erythrocyte sedimentation rate; *HAQ-DI* Health Assessment Questionnaire-Disability Index; *mTSS* modified Total Sharp Score; *RF* rheumatoid factor; *SD* standard deviation

^a^Time from RA diagnosis

^b^Mean for ACPA, RF, and biomarkers was calculated as the geometric mean

### Baricitinib induced a drop in C1M, C3M, and C4M compared to the placebo

All biomarkers were within the detectable range at each time point. Mean levels of C1M, C3M, and C4M at baseline were at the higher end or above the normal healthy control ranges (Table 1 and Table S1), indicating ongoing synovial inflammation in patients with RA. Baricitinib induced a significant drop in C1M, C3M, and C4M compared to placebo for both doses at both time points and for CTX-I for 4 mg at week 12 (Fig. 1). Neither C2M nor osteocalcin changed significantly as a result of baricitinib treatment compared to placebo (*p* > 0.05 for all dose comparison at weeks 4 and 12). While baricitinib did not significantly affect osteocalcin, the ratio between CTX-I/osteocalcin (as an overall measure of bone turnover) showed a significant decrease in the bone turnover ratio for 4 mg at week 12 (percent difference − 16.7, *p* < 0.01); the decrease for the 2-mg dose did not reach the statistical significance. Both doses of baricitinib induced a significant decrease in CRP versus placebo at week 4 (percent difference − 55.4 for 2 mg and − 54.8 for 4 mg; *p* < 0.001) and week 12 (percent difference − 55.2 for 2 mg and − 62.6 for 4 mg; *p* < 0.001).

### Biomarker decrease is associated with improvement in clinical outcome measures

**Fig. 2:**
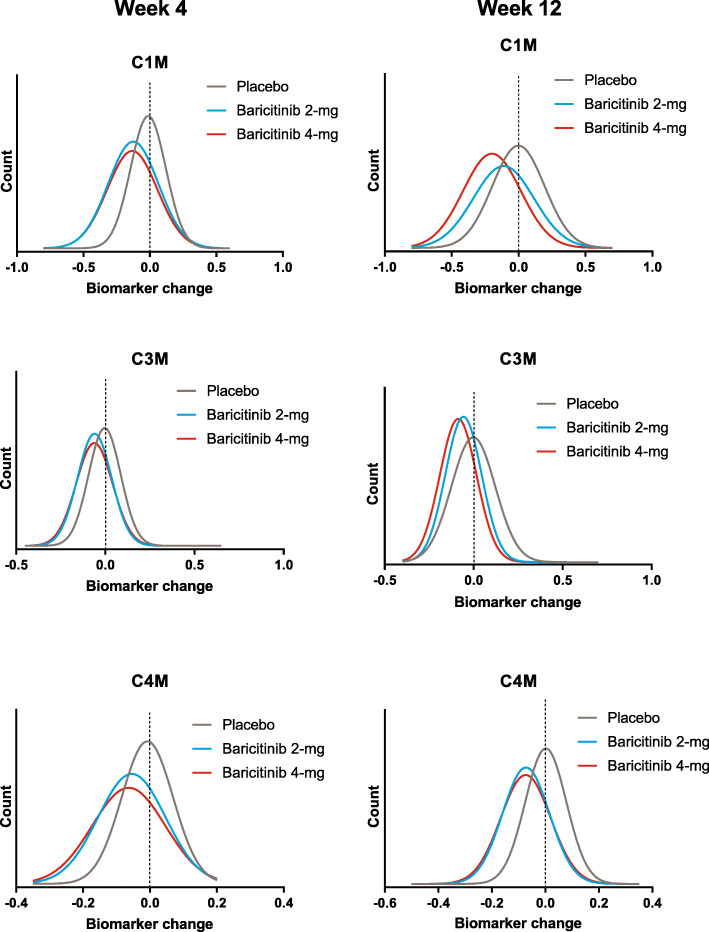
Density plots of changes from baseline to weeks 4, 12 for C1M, C3M, and C4M

**Fig. 3:**
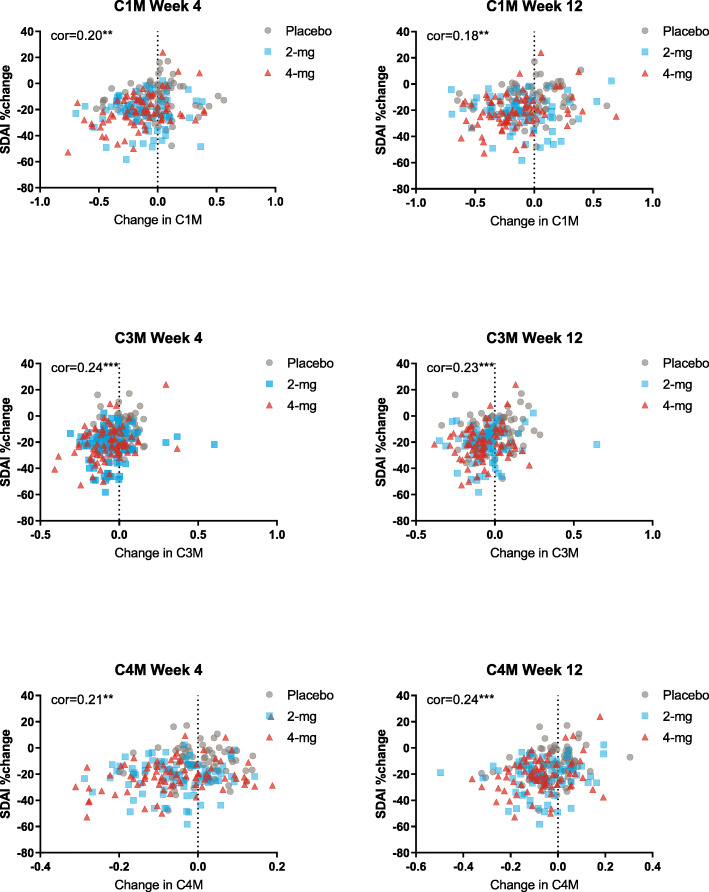
Correlation between change in biomarkers C1M, C3M, and C4M and change in SDAI scores. SDAI, Simplified Disease Activity Index. *p ≤ 0.05; **p ≤ 0.01; ***p ≤ 0.001

While we observed a mean overall decrease in several biomarkers as a result of baricitinib treatment (Fig. 1), we also evaluated the individual absolute changes of these biomarkers at weeks 4 and 12, compared to their baseline values. Figure 2 shows the distribution of absolute change of all patients in each of the 3 treatment groups for C1M, C3M, and C4M at weeks 4 and 12. On average, the biomarkers in the placebo group did not change, as evidenced by the fact that the peak of the curve is at zero (no change). Likewise, it can be seen that about half of the patients receiving placebo showed some decrease in any given biomarker, while an increase was observed in the other half of the patients. In contrast, the peak of the distribution curves shifted to the left in patients treated with baricitinib, indicating that a decrease in biomarker was observed in most patients in the baricitinib groups (Fig. 2). Although clinical improvement in the baricitinib treatment arms was significantly greater compared to placebo, some improvement occurred in patients in the placebo group as well [13]. To determine whether these biomarkers were associated with clinical benefit, we considered changes in biomarkers together with changes in several clinical endpoints across all treatment arms. Correlation analysis showed that a decrease in biomarkers related to synovial inflammation (C1M, C3M, C4M) was associated with clinical improvement based on SDAI at weeks 4 and 12 (Fig. 3). Similar findings were obtained when we considered several other clinical endpoints, including Hybrid ACR, CDAI, HAQDI, and DAS28-ESR, but no relationship was observed with the total number of tender or swollen joint counts or when looking at C2M and CTX-I (Table 2).

**Table 2:** Correlation between clinical outcomes and change in biomarkers at week 12 for patients in all treatment groups

	C1M	C2M	C3M	C4M	CTX-I	CRP
Hybrid ACR	− 0.33***	− 0.06	− 0.31***	− 0.38***	− 0.13	− 0.44***
CDAI	0.1	0.05	0.19**	0.18**	0.16*	0.18**
SDAI	0.18**	0.04	0.23***	0.24***	0.15*	0.25***
HAQ-DI	0.24 ***	− 0.02	0.21**	0.33***	0.09	0.28***
DAS28-ESR	0.35***	0	0.34***	0.35***	0.14*	0.43***
SJC66	− 0.05	0.02	0.15*	0.11	0.1	0.01
TJC68	0.06	0.09	0.13*	0.1	0.1	0.08

Numbers represent Spearman’s rank correlation coefficient value

*ACR* American College of Rheumatology; *C1M* metalloproteinase-derived fragments of type I, of type II (C2M), type III (C3M), and type IV (C4M) collagen; *CDAI* Clinical Disease Activity Index; *CRP* C-reactive protein; *CTX-I* C-terminal telopeptide of type I collagen; *DAS28* Disease Activity Score 28 joints; *ESR* erythrocyte sedimentation rate; *HAQ-DI* Health Assessment Questionnaire-Disability Index; *SDAI* Simplified Disease Activity Index; *SJC66* swollen joint count of 66 joints; *TJC68* tender joint count of 68 joints

*p value ≤ 0.05; **p value ≤ 0.01; ***p value ≤ 0.001

**Fig. 4:**
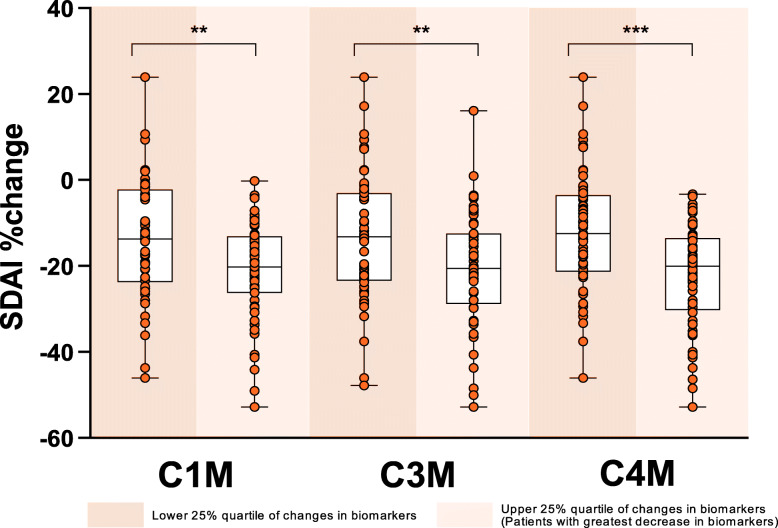
Change in SDAI scores in the lower 25% and upper 25% quartiles of changes in biomarkers C1M, C3M, and C4M at week 12. Patients with the greatest decrease in biomarkers (upper 25% quartile) had significantly greater improvement in SDAI scores compared to those with less of a decrease or possibly an increase in biomarkers. *p ≤ 0.05; **p ≤ 0.01; ***p ≤ 0.001 upper quartile versus lower quartile for percent improvement in SDAI score based on analysis of variance comparison. SDAI, Simplified Disease Activity Index

From these correlation analyses, we further considered the extreme quartiles for each biomarker and compared their change in SDAI clinical score. As expected, patients who had the greatest decrease in C1M (*p* ≤ 0.01), C3M (*p* ≤ 0.01), or C4M (*p* ≤ 0.001) had significantly greater improvement in SDAI scores, compared to patients in the quartile that had less of a decrease, or possibly an increase in each of these markers (Fig. 4). Similar results were seen for other clinical endpoints, including DAS28-ESR, DAS28-CRP, HAQDI, and hybrid ACR (Fig. S1). Finally, we considered the extreme quartiles of each biomarker (lower 25% and upper 25%) and their association with treatment response by calculating the odds ratio of ACR response. In alignment with the continuous clinical variables, patients with the largest decrease in biomarkers had significantly increased odds ratio (defined as *p* value < 0.05) of attaining clinical response for ACR20, ACR50, and ACR70 (Table S2).

When examining radiographic scores in the extreme quartiles (lower and upper 25%), C2M was statistically significantly associated with joint space narrowing, mTSS, and erosion scores (Fig. S2). Similarly, CTX-I was associated with both erosion score and mTSS progression (Fig. S2). However, the number of patients with radiographic progression was low [18], which may explain why we did not see an effect in the systemic levels of the cartilage degradation marker C2M when looking at the whole patient population.

While we did see significant relationships between changes in several biomarkers and clinically relevant endpoints, the absolute value of each of these biomarkers at baseline did not predict clinical improvement (data not shown).

## Discussion

This 12-week substudy of the RA-BUILD clinical trial investigated 8 biomarkers of joint tissue turnover and inflammation in patients with active RA who had inadequate response or intolerance to csDMARDs. Biomarkers consisted of a set of novel neo-epitope biomarkers released during tissue processing, reflecting pathological events in the inflamed tissue, as well as the established inflammation marker CRP. Also included in the analysis were the established bone resorption marker CTX-I and bone formation marker osteocalcin. We characterized the biomarker profiles in response to either 2- or 4-mg baricitinib or placebo and showed a significant reduction in C1M, C3M, and C4M, the biomarkers associated with tissue remodeling of the synovium, in patients receiving both the 2-mg and 4-mg doses of baricitinib, compared to the placebo group. These results are in line with a smaller prospective study that included patients receiving another JAK inhibitor, tofacitinib, where C1M, C3M, and C4M levels were also found to be decreased after 48–60 weeks of treatment compared to each patient’s baseline [19]. Our study is the first randomized, placebo-controlled clinical trial to demonstrate rapid and sustained changes in a soft joint tissue turnover in patients with RA as a result of being treated with a JAK inhibitor.

The reduction of the biomarkers associated with tissue remodeling of the synovium found in this study is in line with the pathogenesis of RA, which likely begins with inflammation in the synovial tissue. Although these interstitial collagens type I and III are expressed in multiple tissues throughout the body, preclinical studies have shown a specific association with tissue turnover in synovial membranes. In an ex vivo synovial membrane model, both C1M and C3M are released upon cytokine challenge and synovitis and inhibited by anti-inflammatory inhibitors such as the syk inhibitor fostamatinib [20]. Type IV collagen is the major constituent of the basement membrane [21], and C4M has been linked to remodeling of the basement membrane in animal models of liver fibrosis [9]. Clinically, C4M has been associated with increased mortality in patients with atherosclerosis, suggesting an association with endothelial remodeling. Although the synovial membrane lacks a regular basement membrane, type IV collagen is found in the intercellular synovial lining and in vascular basement membranes of the normal lining layer [21, 22]. In addition, several studies have argued that a majority of fragments of type IV collagen originate from the RA synovium, showing that fragments are more abundant in patients with active RA [8, 23]. In RA specifically, several studies have investigated the association of biomarkers of collagen tissue turnover with treatment response, disease activity, and progression. Serum C2M, C3M, and C4M are increased in patients with RA compared to healthy donors [8, 24], in line with the observation in the current study that patients’ levels of these markers are in the upper part of the normal range for healthy controls as indicated by the individual assay. In clinical studies investigating the effect of the IL-6 receptor inhibitor tocilizumab, C1M, C3M, and C4M were associated with disease activity and response to treatment, and early reductions were predictive of a treatment response [16, 19]. C1M is associated with bone changes. Baseline C1M levels have been shown to be associated with structural progression measured by mTSS, indicating that C1M is also a marker of bone turnover [25, 26]. In the current study, a reduction in circulating biomarkers associated with tissue destruction and synovial inflammation in patients with RA was observed in baricitinib-treated patients, suggesting that baricitinib inhibits key pathological processes at the site of inflammation in RA.

While markers of the synovial tissue inflammation were reduced, baricitinib showed no effect on C2M. This indicates that baricitinib treatment, in the timespan of the current study, provided limited modulation of cartilage turnover. C2M is a type II collagen fragment released from hyaline cartilage upon proteolytic cleavage by MMPs [27]. C2M is increased in patients with RA compared to healthy controls [24] and can be modulated by anti-inflammatory treatment [17, 20]. Surprisingly, in the current study, we observed no effect on C2M. A possible explanation for the lack of effect may be that cartilage is a low turnover tissue and the limited study timeframe may be too short to allow changes to occur.

CRP levels in the blood decreased significantly upon baricitinib administration in line with previous studies showing a dramatic reduction in the acute phase reactant upon inhibition of inflammatory pathways [19]. JAK1/2 is downstream of the IL-6R and as such it is expected that CRP (as a proxy for IL-6 activity) would decrease. Moreover, patients in this trial were selected as having elevated CRP (≥ 3.6 mg/L), whereas no selection for elevated levels of the tissue-specific markers was made.

An important finding in the current study was that both baseline and change in biomarkers with baricitinib treatment were associated with disease activity parameters and clinical improvement. This clearly links quantitative tissue changes directly with the clinical outcome and shows that biomarkers of tissue remodeling may be used to characterize disease activity and response to treatment.

The limitations of this study include a limited sample size as only a subpopulation of patients from the full RA-BUILD study were included in these analyses. Additionally, the analysis is over a relatively short period of time (12 weeks), unlike previous biomarker studies that had longer follow-up (up to 52 weeks; 14); however, patients in the current study could have received rescue treatment at week 16 and would not have been on their randomized treatment at the time of analysis if it had gone beyond 12 weeks.

Additionally, we would like to see an early reduction in markers be predictive of long-term clinical benefit, more specifically to assess whether changes in biomarkers at week 4 were associated with clinical outcomes at week 12. Improvement in soft tissue biomarkers (C1M, C3M, C4M) at week 4 indeed predicted clinical outcome measures (ACR scores) at week 12.

Baricitinib is approved in many countries for treatment of patients with moderate to severe RA with inadequate response to conventional and/or biologic DMARDs. The current analysis investigates the use in csDMARD inadequate responders, but not biological DMARD naïve patients. The fact that we did not observe any statistically significant difference in changes in biomarkers between the two doses suggests that 2 mg is sufficient for benefit in joint tissue turnover in biological DMARD naïve patients. The current study did not analyze the difference between 2-mg and 4-mg doses in TNFi inadequate responders. Therefore, these biomarkers should be tested in both dosage arms in a biological DMARD inadequate responder study to expand the current findings.

## Conclusions

In conclusion, this study shows that baricitinib reduced circulating biomarkers associated with joint tissue destruction, suggesting it inhibits key pathological processes in RA. Importantly, the decrease in biomarkers was associated with clinical improvement, as measured by a number of disease activity measures including SDAI, CDAI, HAQ-DI, hybrid ACR, and DAS28-ESR.

## Supplementary information

**Additional file 1 **: **Table S1**. Upper and lower levels of quantification and normal ranges for each assay measured.

**Additional file 2 **: **Table S2**. Odds ratio for ACR response at Week 12 with biomarker changes at Weeks 4 and 12 measured by patients being in the lower 25% versus the upper 25% quartile for change in biomarker for patients in all treatment groups.

**Additional file 3 **: **Figure S1**. Change in clinical scores (DAS28-ESR, DAS28-CRP, HAQ-DI, and hybrid ACR) in the lower 25% and upper 25% quartiles of changes in biomarkers C1M, C3M, and C4M at Week 12. Patients with the greatest decrease in biomarkers (upper 25% quartile) had significantly greater improvement in clinical scores compared to those with less of a decrease, or possibly an increase in biomarkers. *p≤0.05; **p≤0.01; ***p≤0.001 upper quartile versus lower quartile for percent improvement in clinical score based on analysis of variance comparison. ACR, American College of Rheumatology; DAS28-ESR, Disease Activity Score 28 joints with erythrocyte sedimentation rate; DAS28-CRP, Disease Activity Score 28 joints with C-reactive protein.

**Additional file 4 **: **Figure S2.** Change in radiographic scores in the lower 25% and upper 25% quartiles of changes in biomarkers C1M, C3M, C4M, C2M, CTX-I and osteocalcin at Week 12. *p≤0.05; **p≤0.01; ***p≤0.001 upper quartile versus lower quartile for percent improvement in radiographic score based on analysis of variance comparison. mTSS, modified Total Sharp Score.

## Data Availability

Lilly provides access to all individual participant data collected during the trial, after anonymization, with the exception of pharmacokinetic or genetic data. Data are available to request 6 months after the indication studied has been approved in the US and EU and after primary publication acceptance, whichever is later. No expiration date of data requests is currently set once data are made available. Access is provided after a proposal has been approved by an independent review committee identified for this purpose and after receipt of a signed data sharing agreement. Data and documents, including the study protocol, statistical analysis plan, clinical study report, and blank or annotated case report forms, will be provided in a secure data sharing environment. For details on submitting a request, see the instructions provided at www.vivli.org.

## Abbreviations

ACPA: Anti-citrullinated peptide antibodies
ACR: American College of Rheumatology
ACR20/50/70% response: American College of Rheumatology (ACR) 20%/50%/70% response rate
CDAI: Clinical Disease Activity Index
CRP: C-reactive protein
csDMARD: Conventional synthetic disease-modifying antirheumatic drugs
CTX-I: C-terminal telopeptide of type I collagen
DAS28: Disease Activity Score for 28 joints
DMARD: Disease-modifying antirheumatic drug
ESR: Erythrocyte sedimentation rate
HAQ-DI: Health Assessment Questionnaire-Disability Index
hsCRP: High sensitivity C-reactive protein
IL: Interleukin
IFNγ: Interferon gamma
JAK: Janus kinase
MMP: Matrix metalloproteinase
mTSS: Modified Total Sharp Score
RA: Rheumatoid arthritis
RF: Rheumatoid factor
SJC66: Swollen joint count of 66 joints
SDAI: Simplified Disease Activity Index
TJC68: Tender joint count of 68 joints
TNFi: Tumor necrosis factor inhibitor
