# Supplementary material for: The Janus kinase 1/2 inhibitor baricitinib reduces biomarkers of joint destruction in moderate to severe rheumatoid arthritis
Source: Arthritis Res Ther. 2020 Oct 12;22:235. doi: 10.1186/s13075-020-02340-7 (PMC7552555; doi:10.1186/s13075-020-02340-7)
Supplement: Supplementary file 1 — Additional file 1 : Table S1. Upper and lower levels of quantification and normal ranges for each assay measured. [file 13075_2020_2340_MOESM1_ESM.docx]

**Additional material: Table S1.** Upper and lower levels of quantification and normal ranges for each assay measured

| **Biomarker** | **ULOQ (ng/ml)** | **LLOQ (ng/ml)** | **Normal range (ng/ml) in healthy subjects** | **Range (ng/ml) at baseline in patients in this study** |
| --- | --- | --- | --- | --- |
| C1M^1^ | 150.6 | 6.6 | 18.0 - 41.8* | 13.2 – 252.9 |
| C2M^1^ | 1.78 | 0.13 | 0.16-0.94* | 0.13 – 1.15 |
| C3M^1^ | 80.2 | 2.8 | 5.7 - 13.6* | 5.2 – 69.1 |
| C4M^1^ | 118.4 | 3.1 | 14.1 - 37.6* | 9.7-110.0 |
| CTX-I^2-5^ | 2.880 | 0.010 | Female: 0.104 - 1.008  Male: 0.016 - 0.704 | Female: 0.032 – 1.1  Male: 0.014 – 1.15 |
| Osteocalcin^5,6^ | 209.6 | 0.5 | 15.0 - 46.0 | 2.19 – 67.86 |

*Postmenopausal women
C1M, metalloproteinase-derived fragments of type I; of type II (C2M); type III (C3M); and type IV (C4M) collagen; CTX-I, C-terminal telopeptide of type I collagen; LLOQ, lower limit of quantification; ULOQ, upper limit of quantification

References:

1. Hušáková M, Bay-Jensen AC, Forejtová Š, Zegzulková K, Tomčík M, Gregová M, Bubová K, Hořínková J, Gatterová J, Pavelka K, Siebuhr AS. Metabolites of type I, II, III, and IV collagen may serve as markers of disease activity in axial spondyloarthritis. Sci Rep. 2019 Aug 2;9(1):11218. doi: 10.1038/s41598-019-47502-z. Erratum in: Sci Rep. 2020 Aug 19;10(1):14133. PMID: 31375691; PMCID: PMC6677742.
2. Christgau S, Bitsch-Jensen O, Hanover Bjarnason N, Gamwell Henriksen E, Qvist P, Alexandersen P, Bang Henriksen D. Serum CrossLaps for monitoring the response in individuals undergoing antiresorptive therapy. Bone. 2000 May;26(5):505-11. doi: 10.1016/S8756-3282(00)00248-9. PMID: 10773591.
3. Garnero P, Borel O, Delmas PD. Evaluation of a fully automated serum assay for C-terminal cross-linking telopeptide of type I collagen in osteoporosis. Clin Chem. 2001 Apr;47(4):694-702. PMID: 11274020.
4. Delmas PD, Eastell R, Garnero P, Seibel MJ, Stepan J; Committee of Scientific Advisors of the International Osteoporosis Foundation]. The use of biochemical markers of bone turnover in osteoporosis. Committee of Scientific Advisors of the International Osteoporosis Foundation. Osteoporos Int. 2000;11 Suppl 6:S2-17. doi: 10.1007/s001980070002. PMID: 11193237.
5. Hu WW, Zhang Z, He JW, Fu WZ, Wang C, Zhang H, Yue H, Gu JM, Zhang ZL. Establishing reference intervals for bone turnover markers in the healthy shanghai population and the relationship with bone mineral density in postmenopausal women. Int J Endocrinol. 2013;2013:513925. doi: 10.1155/2013/513925. Epub 2013 Feb 27. PMID: 23533403; PMCID: PMC3600195.
6. Hannemann A, Friedrich N, Spielhagen C, Rettig R, Ittermann T, Nauck M, Wallaschofski H. Reference intervals for serum osteocalcin concentrations in adult men and women from the study of health in Pomerania. BMC Endocr Disord. 2013 Mar 13;13:11. doi: 10.1186/1472-6823-13-11. PMID: 23497286; PMCID: PMC3606466.
