# Supplementary material for: The Janus kinase 1/2 inhibitor baricitinib reduces biomarkers of joint destruction in moderate to severe rheumatoid arthritis
Source: Arthritis Res Ther. 2020 Oct 12;22:235. doi: 10.1186/s13075-020-02340-7 (PMC7552555; doi:10.1186/s13075-020-02340-7)
Supplement: Supplementary file 2 — Additional file 2 : Table S2. Odds ratio for ACR response at Week 12 with biomarker changes at Weeks 4 and 12 measured by patients being in the lower 25% versus the upper 25% quartile for change in biomarker for patients in all treatment groups. [file 13075_2020_2340_MOESM2_ESM.docx]

**Additional material: Table S2**. Odds ratio for ACR response at Week 12 with biomarker changes at Weeks 4 and 12 measured by patients being in the lower 25% versus the upper 25% quartile for change in biomarker for patients in all treatment groups

|  |  | **C1M** | | **C3M** | | **C4M** | | **CRP** | |
| --- | --- | --- | --- | --- | --- | --- | --- | --- | --- |
|  |  | Week 4 | Week 12 | Week 4 | Week 12 | Week 4 | Week 12 | Week 4 | Week 12 |
| ACR20 | OR (95%CI)  p-value | 3.03 (1.4-6.7)  0.006 | 4.8 (2.1-10.7)  <0.001 | 2.4 (1.1-5.2)  0.036 | 2.4 (1.1-5.2)  0.025 | 2.7 (1.3-5.6)  0.015 | 4.4 (2.0-9.5)  <0.001 | 5.4 (2.5-12.0)  <0.001 | 4.4 (2.0-9.6)  <0.001 |
| ACR50 | OR (95%CI)  p-value | 2.2 (1.0-5.0)  0.069 | 5.1 (2.0-13.3)  <0.001 | 2.7 (1.1-6.5)  0.034 | 3.15 (1.3-7.7)  0.011 | 2.0 (0.9-4.3)  0.116 | 4.5 (1.7-11.5)  0.002 | 3.7 (1.6-8.6)  0.002 | 4.2 (1.8-9.9)  <0.001 |
| ACR70 | OR (95%CI)  p-value | 2.1 (0.7-6.2)  0.19 | 4.3 (1.1-16.5)  0.042 | 4.9 (1.3-18.4)  0.013 | 8.4 (1.8-39.3)  0.002 | 2.5 (0.9-6.6)  0.097 | 8.9 (1.9-41.3)  0.002 | 6.0 (1.6-22.3)  0.003 | 9.0 (1.9-41.7)  0.001 |

ACR20/50/70, 20%, 50%, 70% American College of Rheumatology response rate; CI, confidence interval; C1M, metalloproteinase-derived fragments of type I; type III (C3M); and type IV (C4M) collagen; CDAI, Clinical Disease Activity Index; CRP, C-reactive protein; OR, odds ratio.
