# Supplementary material for: The Janus kinase 1/2 inhibitor baricitinib reduces biomarkers of joint destruction in moderate to severe rheumatoid arthritis
Source: Arthritis Res Ther. 2020 Oct 12;22:235. doi: 10.1186/s13075-020-02340-7 (PMC7552555; doi:10.1186/s13075-020-02340-7)
Supplement: Supplementary file 3 — Additional file 3 : Figure S1. Change in clinical scores (DAS28-ESR, DAS28-CRP, HAQ-DI, and hybrid ACR) in the lower 25% and upper 25% quartiles of changes in biomarkers C1M, C3M, and C4M at Week 12. Patients with the greatest decrease in biomarkers (upper 25% quartile) had significantly greater improvement in clinical scores compared to those with less of a decrease, or possibly an increase in biomarkers. *p≤0.05; **p≤0.01; ***p≤0.001 upper quartile versus lower quartile for percent improvement in clinical score based on analysis of variance comparison. ACR, American College of Rheumatology; DAS28-ESR, Disease Activity Score 28 joints with erythrocyte sedimentation rate; DAS28-CRP, Disease Activity Score 28 joints with C-reactive protein. [file 13075_2020_2340_MOESM3_ESM.docx]

**Figure S1.** Change in clinical scores (DAS28-ESR, DAS28-CRP, HAQ-DI, and hybrid ACR) in the lower 25% and upper 25% quartiles of changes in biomarkers C1M, C3M, and C4M at week 12. Patients with the greatest decrease in biomarkers (upper 25% quartile) had significantly greater improvement in clinical scores compared to those with less of a decrease, or possibly an increase in biomarkers. *p≤0.05; **p≤0.01; ***p≤0.001 upper quartile versus lower quartile for percent improvement in clinical score based on analysis of variance comparison. ACR, American College of Rheumatology; DAS28-ESR, Disease Activity Score 28 joints with erythrocyte sedimentation rate; DAS28-CRP, Disease Activity Score 28 joints with C-reactive protein.
