# Supplementary material for: The Janus kinase 1/2 inhibitor baricitinib reduces biomarkers of joint destruction in moderate to severe rheumatoid arthritis
Source: Arthritis Res Ther. 2020 Oct 12;22:235. doi: 10.1186/s13075-020-02340-7 (PMC7552555; doi:10.1186/s13075-020-02340-7)
Supplement: Supplementary file 4 — Additional file 4 : Figure S2. Change in radiographic scores in the lower 25% and upper 25% quartiles of changes in biomarkers C1M, C3M, C4M, C2M, CTX-I and osteocalcin at Week 12. *p≤0.05; **p≤0.01; ***p≤0.001 upper quartile versus lower quartile for percent improvement in radiographic score based on analysis of variance comparison. mTSS, modified Total Sharp Score. [file 13075_2020_2340_MOESM4_ESM.docx]

**Figure S2**. Change in radiographic scores in the lower 25% and upper 25% quartiles of changes in biomarkers C1M, C3M, C4M, C2M, CTX-I, and osteocalcin at Week 12. *p≤0.05; **p≤0.01; ***p≤0.001 upper quartile versus lower quartile for percent improvement in radiographic score based on analysis of variance comparison. mTSS, modified Total Sharp Score
